# Lower basal and postprandial muscle protein synthesis after 2 weeks single‐leg immobilization in older men: No protective effect of anti‐inflammatory medication

**DOI:** 10.14814/phy2.15958

**Published:** 2024-02-26

**Authors:** K. Dideriksen, S. Reitelseder, A. P. Boesen, M. Zillmer, J. Agergaard, M. Kjaer, L. Holm

**Affiliations:** ^1^ Institute of Sports Medicine Copenhagen, Department of Orthopedic Surgery Copenhagen University Hospital—Bispebjerg and Frederiksberg Copenhagen Denmark; ^2^ Center for Healthy Aging, Department of Clinical Medicine University of Copenhagen Copenhagen Denmark; ^3^ Faculty of Health and Medical Sciences, Institute of Biomedical Sciences University of Copenhagen Copenhagen Denmark

**Keywords:** anabolic resistance, ibuprofen, muscle disuse, muscle inactivity, muscle protein synthesis signaling, myofibrillar FSR

## Abstract

Muscle inactivity may reduce basal and postprandial muscle protein synthesis (MPS) rates in humans. Anti‐inflammatory treatment alleviates the MPS impairments in younger individuals. The present study explored the influence of nonsteroidal anti‐inflammatory drugs (NSAIDs) upon MPS during a period of inactivity in older humans. Eighteen men (age 60–80 years) were allocated to ibuprofen (1200 mg/day, Ibu) or control (Plc) groups. One lower limb was cast immobilized for 2 weeks. Postabsorptive and postprandial MPS was measured before and after the immobilization by L‐[*ring*‐^13^C_6_]‐phenylalanine infusion. The protein expression of select anabolic signaling molecules was investigated by western blot. Basal (0.038 ± 0.002%/h and 0.039 ± 0.005%/h, Plc and Ibu, respectively) and postprandial (0.064 ± 0.004%/h and 0.067 ± 0.010%/h, Plc and Ibu, respectively) MPS rate were higher pre‐immobilization compared to basal (0.019 ± 0.005%/h and 0.020 ± 0.010%/h, Plc and Ibu, respectively) and postprandial (0.033 ± 0.005%/h and 0.037 ± 0.006%/h, Plc and Ibu, respectively) MPS rate post‐immobilization (*p* < 0.001). NSAID treatment did not affect the suppression of MPS (*p* > 0.05). The anabolic signaling were in general reduced after immobilization (*p* < 0.05). These changes were unaffected by NSAID treatment (*p* > 0.05). Basal and postprandial MPS dropped markedly after 2 weeks of lower limb immobilization. NSAID treatment neither influenced the reduction in MPS nor the anabolic signaling after immobilization in healthy older individuals.

## 1 INTRODUCTION

Studies have shown that bedrest and limb immobilization decrease the basal postabsorptive muscle protein synthesis (MPS) rate (de Boer et al., 2007; Ferrando et al., 1996, 1997; Glover et al., 2008; Kortebein et al., 2007; Wall et al., 2016). In addition, reductions in the anabolic sensitivity to amino acids (anabolic resistance) has been demonstrated after muscle inactivity, both in the form of reduced use (Breen et al., 2013), bedrest (Biolo et al., 2004; Drummond et al., 2012; Tanner et al., 2015), and limb immobilization (Glover et al., 2008; Wall et al., 2013, 2016). Such changes in rates and responsiveness of MPS may be partly responsible for the well‐known net loss of muscle mass during periods of muscle inactivity (Breen et al., 2013; de Boer et al., 2007; Glover et al., 2008; Tanner et al., 2015).

In older individuals, increased systemic inflammation has been demonstrated after 2 weeks of bed rest (Jurdana et al., 2015) as well as after 2 weeks of a daily step‐reduction of ~76% (Breen et al., 2013). The presence of such slightly increased inflammation (termed low‐grade inflammation) induced by external conditions like muscle inactivity may reduce MPS and thereby be partly responsible for the loss of muscle mass and strength in older individuals. It is possible that the whole‐body inactivity models (Breen et al., 2013; Jurdana et al., 2015) may have a more pronounced systemic impact when compared to a local limb immobilization model though.

In old rats with low‐grade inflammation, the blunted postprandial stimulation of MPS (Balage et al., 2010) can be prevented with ibuprofen treatment, a nonsteroidal anti‐inflammatory drug (NSAID) (Rieu et al., 2009). Moreover, anti‐inflammatory treatment (dietary fish oil supplementation) prior to and during 10 days of immobilization in healthy adult rats, has been shown to attenuate muscle catabolism (You et al., 2010). This muscle‐preserving effect was partly achieved by upholding Akt and p70‐S6K1 kinase activity (You et al., 2010). Signaling through the mTORC1 pathway is key in regulation of MPS and has shown to be impaired after bed‐rest (Drummond et al., 2012; Tanner et al., 2015). Thus, upholding such anabolic signaling during muscle inactivation with anti‐inflammatory treatment could alleviate the negative impact on MPS.

The aim of this study was to investigate the influence of NSAID supplementation on the overnight post‐absorptive (basal) MPS and the postprandial MPS (after whey protein feeding) before and immediately after 2 weeks of single‐leg immobilization, as well as associated molecular regulation (protein signaling) in healthy older men.

We hypothesized that basal myofibrillar fractional synthesis rate (FSR), as well as postprandial myofibrillar FSR would be reduced after the immobilization period. Furthermore, it was hypothesized that treatment with NSAID would preserve postprandial myofibrillar FSR responsiveness (i.e., lessen anabolic resistance) after immobilization.

## 2 METHODS

### 2.1 Study design

Eighteen healthy, old men (age: 60–80 years, BMI: 20–30 kg/m^2^) were recruited through newspaper advertisements. The included participants were screened free of cancer, metabolic, cardiac, and neurological diseases, were nonsmokers and recreationally physically active, and had not taken part in any form of strenuous endurance or resistance training prior to trial participation. Moreover, participants were instructed not to take any kind of analgesic medication at least 2 weeks before the beginning of the study. Finally, all participants gave their written informed consent before being enrolled in the experiment that was approved by the Copenhagen Ethics Committee (H‐1‐2010‐007) and conformed to the Helsinki Declaration.

The present investigation was part of a study that investigated the effect of ibuprofen treatment on muscle mass and strength adaptation during 2 weeks of lower limb immobilization and 6 weeks of subsequent retraining (Dideriksen et al., 2016). Furthermore, the participant characteristic data of included individuals have been used in previous studies (Boesen et al., 2014; Dideriksen et al., 2016, 2017, 2020), whereas the MPS and anabolic signaling outcome data have not been reported previously.

**FIGURE 1:**
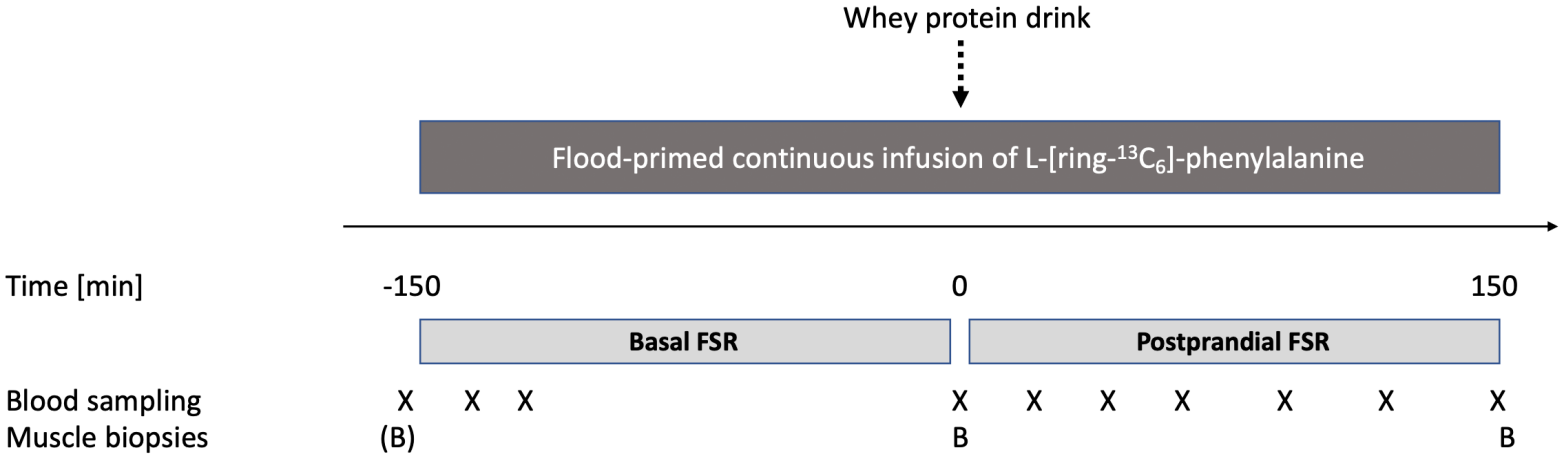
Study infusion protocol. Eighteen older men completed 2 weeks of immobilization, with either ibuprofen (2 × 600 mg/day) (*n* = 7) or placebo (*n* = 11) administration, and whey protein ingestion (2 × 20 g/day). Experimental days were conducted for measurement of muscle myofibrillar fractional synthesis rates (FSR) before and after 2 weeks of immobilization. Throughout the infusion periods, blood samples (x) and muscle biopsies (B) were collected. A background biopsy ((B)) before time point −150 min were obtained only after the immobilization period.

The study intervention included 2 weeks of immobilization and whey protein ingestion, with either ibuprofen or placebo administration. Two acute experimental days (Figure 1), including tracer infusion and whey protein ingestion, were conducted; one before and one after the immobilization period.

### 2.2 Ibuprofen treatment and protein supplementation

The included individuals were allocated to one of two groups receiving either ibuprofen (Ibu) or placebo (Plc) in a randomized double‐blinded fashion. As reported in previous studies (Dideriksen et al., 2016, 2017, 2020), eight participants were allocated to the Ibu group receiving 2 × 600 mg ibuprofen/day. However, one participant was excluded from the present report since he did not fulfill the requirements for the first infusion trial. Therefore, the groups ended as Ibu, *n* = 7 and Plc, *n* = 11. As previously described (Dideriksen et al., 2016), the Plc group consisted of participants from two studies. The first group consisting of 5 participants were included in a previous study (Boesen et al., 2014) where they were randomized to receive placebo injections in a double‐blinded fashion (saline or growth hormone), whereas the second group consisting of 6 participants were enrolled directly in the present study where they were randomized to receive placebo tablets (potato starch and lactose monohydrate) in a double‐blinded fashion. Even though the Plc group consisted of two groups of participants, all subjects were continuously enrolled and randomized by the envelope method. Though ibuprofen works within hours (Dewland et al., 2009; Rainsford, 2009), the ibuprofen treatment started 2 days before immobilization (immediately after completion of the first tracer infusion trial) to ensure that the effect of ibuprofen was obtained from the beginning of the immobilization period. All individuals were instructed to take their daily tablets at the same time every morning and evening together with a meal and to return the empty packages. Furthermore, participants were not allowed to consume any cyclooxygenase‐inhibiting drugs besides the tablets provided in accordance with the study protocol.

The present study was carried out under free‐living conditions with no measurement of dietary intake during the study period. However, the habitual diet of the participants was assessed before inclusion, and they were encouraged to maintain their habitual diets throughout the study period. Furthermore, participants consumed 2 × 20 g of whey protein (Lacprodan, Arla Foods Ingredients P/S, Viby J, Denmark) each day, to ensure that eventual differences in muscle FSR between groups would not be due to insufficient intake of protein and essential amino acids in one of the groups. Participants consumed the first whey protein drink in the laboratory 2 days before immobilization and were instructed to consume a drink after breakfast and a drink again after lunch on each day.

### 2.3 Unilateral limb immobilization

The immobilization procedure has previously been described in detail and shown to induce substantial quadriceps muscle atrophy in both young and old individuals (Boesen et al., 2013, 2014). Briefly, immobilization was accomplished by a lightweight fiber cast applied from the groin to just proximal to the malleoli, in a 50° position of knee joint flexion (randomly selected limb). Participants were carefully instructed to avoid any kind of quadriceps muscle contractions of the immobilized leg and to use crutches for locomotion. However, participants were requested to remain physically active during the unilateral immobilization period, although it may cause them some difficulties. Moreover, all participants were treated with acetylsalicylic acid (75 mg/day) during the 2‐week immobilization period to reduce the potential risk of deep venous thrombosis. Even though it has been shown that low‐dose aspirin (corresponding to oral dose of 75–325 mg) can reduce muscle PGE2 production *ex vivo* (Fountain et al., 2020), the *in vivo* anti‐inflammatory effect of such a low dose remains unknown.

### 2.4 Baseline measurements

At baseline, lean body mass (LBM) was determined by Dual‐energy x‐ray absorptiometry scanning (Lunar DPX‐IQ, GE Healthcare, Chalfont St. Giles, UK) for calculation of the tracer infusion rate. During the scan, the individuals were wearing light clothing, no removable metal objects, and placed in a supine position.

### 2.5 Experimental tracer infusion protocol

Experimental days were conducted prior to and immediately after cast removal. Both days included measurement of muscle myofibrillar protein FSR in the overnight fasted and the postprandial state (Figure 1). Participants were instructed to refrain from alcohol and strenuous physical activity, and to follow their normal eating pattern 72 h prior to the experimental day. Furthermore, no intake of caffeine was allowed 24 h before the trial. The trial conducted prior to the immobilization period was completed before the start of NSAID intake (2 days before the beginning of the immobilization period). Participants arrived at the laboratory by car or public transportation after an overnight fast (from 10.00 p.m. the day before the experiment). Participants were placed in a supine position and a catheter was inserted into a antecubital vein on both forearms and a background blood sample was taken. A flood priming bolus of L‐[*ring*‐^13^C_6_]‐phenylalanine (1485 mg of phenylalanine with a tracer‐to‐tracee ratio (TTR) corresponding to 12%) were given over 1–2 min and followed by a continuous infusion of tracer (1.28 mg × kg LBM^−1^ × h^−1^) aiming at 12% TTR arterial enrichment (Holm et al., 2014) to obtain a high analytical sensitivity of the incorporated tracer abundance in the muscle proteins across the relatively short exposure time. Throughout the trial, venous blood samples were collected at time points −160, −135, −120, 0, 20, 40, 60, 90, 120, and 150 min after infusion start into EDTA tubes that were cooled on ice for 10 min followed by centrifugation (10 min at 3970 *g* at 4°C). Plasma was stored at −80°C for measurement of phenylalanine tracer enrichment as well as leucine and insulin concentration.

After the blood sample at 0 min, participants ingested 0.45 g × kg LBM^−1^ whey protein isolate (Lacprodan DI‐9224, Arla Foods Ingredients P/S, Viby J, Denmark) dissolved in water, resulting in individualized drinks with a total content of 25.1–32.9 g protein. To minimize disturbance of isotopic steady state once phenylalanine from the protein drink got absorbed systemically, free L‐[*ring*‐^13^C_6_] phenylalanine tracer was added to the whey protein drink in an amount corresponding to 10% of the total phenylalanine content in the drink, based on the amino acid content in the whey protein provided by Arla Foods Ingredients P/S. Immediately after the whey protein ingestion, and again 150 min post protein intake, a muscle biopsy was obtained from the vastus lateralis muscle using local anesthesia (lidocaine, 1%) and a 5‐mm Bergström needle with suction. The muscle biopsies were cleared of external adipose tissue, connective tissue, blood, and divided in 2 portions for measurement of myofibrillar protein synthesis and anabolic signaling, respectively, before they were frozen in liquid nitrogen and stored at −80°C. At the first experimental day, the muscle biopsies were taken from the limb that was not to be subjected to immobilization, whereas at the second experimental day, the muscle biopsies were taken from the contralateral limb, which had been immobilized. The biopsies from the same limb were placed at least 3 cm apart in a randomized location order to obtain samples unaffected by previous biopsies.

When the infusion trial was repeated on the day of cast removal a background biopsy was taken for measurement of L‐[*ring*‐^13^C_6_]‐phenylalanine abundance in myofibrillar proteins originating from the first infusion trial. The two experimental infusion trials were started out at the same time in the morning to avoid possible effects of circadian fluctuations on outcome parameters.

### 2.6 Plasma sample analyses

Plasma leucine concentration, as well as phenylalanine tracer enrichment, were measured in 200 μL plasma added a known amount of L‐[^13^C_6_]‐leucine (internal standard) as described previously (Holm et al., 2014). Samples were acidified with 1 mL of 50% acidic acid and poured over resin columns (AG 50 W‐X8 resin; Bio‐Rad Laboratories, Hercules, CA) preconditioned with 1 mL of 50% acidic acid. After five washes with Milli‐Q water, the purified amino acids were eluted with 2 × 1 mL of 2 M NH_4_OH into new vials. After being dried down, the purified amino acids were derivatized using *N*‐methyl‐*N*‐(*tert*‐butyldimethylsilyl)trifluoroacetamide +1% *tert*‐butyl‐dimethylchlorosilane (Regis Technologies, Morton Grove, IL) and acetonitrile, with a mixing ratio of 1:1. 1‐μL sample was injected in the programmed temperature vaporization injection mode on to a gas chromatograph and the amino acid derivatives were separated on a CP‐Sil 8 CB capillary column (30 m, 0.32 mm ID; coating, 0.25 μm) (ChromPack; Varian, Palo Alto, CA). Derivatives of the phenylalanine and leucine amino acids were ionized by electron ionization and their mass‐to‐charge abundances were analyzed on a triple‐stage quadrupole‐mass spectrometer (GC‐MS/MS, TSQ Quantum; Thermo Scientific, San Jose, CA) as previously described (Holm et al., 2014).

From background blood samples taken before the first L‐[*ring*‐^13^C_6_]‐phenylalanine infusion trial before immobilization, plasma proteins were precipitated with 500 μL ice‐cold acetone on 75 μL plasma sample. After precipitation, the sample was spun down at 1800 g for 10 min, and the protein pellet was washed in 70% EtOH. Subsequently, the proteins were hydrolyzed by addition of 1 mL 6 M HCl, and left overnight at 110°C.

Plasma insulin concentrations were determined using ELISA kits (#K621911‐2, Dako, Glostrup, Denmark) with intra‐assay CV ≤7.5% and inter‐assay CV < 10%.

### 2.7 Muscle tissue analyses

Muscle specimens of 10–20 mg wet weight were homogenized (Fast‐prep, 120A‐230; Thermo Savant, Holbrook, NY) for 45 s in 1 mL of ice‐cold saline and subsequently spun (4°C, 1600 g, 10 min). The supernatant was removed, and the muscle pellet was added 1 mL homogenization buffer (0.02 M Tris, pH 7.4, 0.15 M NaCl, 2 mM EDTA, 0.5% Triton‐X 100, 0.25 M sucrose), homogenized 4 × 45 s, and left for 3 h at 4°C. After a spin (4°C, 800 g, 20 min), the pellet was added 1 mL homogenization buffer (0.02 M Tris, pH 7.4, 0.15 M NaCl, 2 mM EDTA, 0.5% Triton‐X 100, 0.25 M sucrose), homogenized again for 45 s, and left for 30 min at 4°C. After a spin (4°C, 800 g, 20 min), the supernatant was discarded and 1.5 mL of high‐salt solution (0.7 M KCl, 0.1 M pyrophosphate) was added to pellet, which was again homogenized for 45 s and left overnight at 4°C. After vortex and spinning (4°C, 1600 g, 20 min), the supernatant containing the myofibrillar proteins was added 3.45 mL ethanol (99%) to precipitate proteins, vortexed, and left for 2 h at 4°C. After spinning (4°C, 1600 g, 20 min) supernatant was discarded and 1 mL of 70% ethanol was added to the pellet, which was vortexed and spun (4°C, 1600 g, 20 min), where after the pellet was hydrolyzed overnight in 1 mL of 6 M HCl at 110°C.

The liberated amino acids from the myofibrillar and plasma proteins, respectively, were purified over resin columns (as described above for the plasma free amino acids) and N‐acetyl‐n‐propyl (NAP)‐derivatized. The ^13^C abundance of the NAP‐derivatized phenylalanine compounds originating from the myofibrillar proteins were analyzed using a CP‐Sil 19 CB capillary column (60 m × ID 0.25 mm, coating 0.25 μm) (AgilentJ&W) on a gas chromatograph‐combustion‐isotope ratio mass spectrometer (GC‐C‐IRMS) (Delta Plus XL; Thermo Finnigan, Bremen, Germany) to isolate and measure ^13^C abundance in the muscle tissue protein samples.

### 2.8 Western blot analyses

Muscle samples of 10–25 mg were homogenized using a plastic pestle and fractionated into a cytosolic and nuclear fraction using a commercial fractionation kit (ProteoExtract Subcellular Proteome Extraction Kit, 539790, Merck, Darmstadt, Germany) according to the manufacturer's procedures. Subsequently, protein concentrations on the cytosolic and nuclear fractions were quantified using a commercial micro plate kit (BioRad DC protein micro plate assay, 0113, 0114, 0115, Bio‐Rad Laboratories, Hercules, CA, USA). Data on catabolic targets from the cytosolic and nuclear fractions has previously been published (Dideriksen et al., 2020). Here the cytosolic fraction was used, to quantify anabolic signaling from select targets of the mTORC1‐signaling pathway.

Homogenates of the cytosolic fraction were diluted 1:1 in 2X Sample buffer (1.25 M Tris pH 6.8, 25% v/v Glycerol, 2.5% SDS, 2.5% v/v β‐mercaptoethanol and 0.2% w/v Bromophenol Blue). Samples for loading were heated at 95°C for 5 min prior to loading on SDS‐PAGE gels. Equal amounts of protein from the cytosolic fraction were loaded per well (10 μg) and separated by 7.5% (mTOR and p70‐S6K) or 12% (eEF2, AKT, 4E‐BP1) 26 well TGX Stain‐Free Precast protein gels (#5671025 and #5678024, respectively, Bio‐Rad Laboratories, Hercules, CA, USA). SDS‐PAGE was run for 1 h and 10 min at 150 V in electrophoresis buffer (25 mM Tris‐Base, 190 mM Glycine and 3.5 mM SDS). Subsequently, protein was blotted by wet‐transfer (#170‐3912, Bio‐Rad, Hercules, CA) to low‐fluorescence PVDF membranes (Bio‐Rad, Hercules, CA) for 1 h at 50 V in transfer buffer (10 mM CAPS pH 11.0 and 10% v/v methanol). Membranes were blocked for 30 min at room temperature with 20% v/v Odyssey blocking buffer (Li‐Cor Biosciences, Lincoln, NE). The membranes were then incubated with primary antibodies overnight at 4°C in a solution of 10% v/v Odyssey blocking buffer and 0.1% v/v Tween‐20. The primary antibodies from Cell Signaling Technology, Danvers, MA were total‐mTOR (no. 2983) diluted 1:1000, total‐p70‐S6K1 (no. 2708) diluted 1:1000, p‐p70‐S6K (Thr389; no. 9206) diluted 1:1000, total‐AKT1 (no. 2967) diluted 1:1000, p‐AKT (Thr308; no. 2965) diluted 1:1000, p‐eEF2 (Thr56; no. 2331) diluted 1:2000 and p‐4E‐BP1 (Thr37/46; no. 2855) diluted 1:1000, and from Abcam, Cambridge, MA total‐eEF2 (ab130187) diluted 1:500. Membranes were washed 3 times for 5 min in washing‐buffer (TBS with 0.1% v/v Tween 20, pH 7.4) and incubated for 1 h at room temperature in fluorescent secondary antibody; anti‐mouse, Alexa 680 (no. A21057, Invitrogen, Carlsbad, CA) diluted 1:10000, and anti‐rabbit, DyLight 800 (no. 35571, Pierce Biotechnology, Rockford, IL) diluted 1:10000. Subsequently, the membranes were washed and visualized (Odyssey Infrared Imaging System; Li‐Cor Biosciences, Lincoln, NE). Band intensities were quantified using ImageJ (National Institutes of Health, Bethesda, MD). Ponceau‐S staining of the membranes was used to control that equal amount of protein had been loaded onto the gels before SDS‐PAGE. Membranes were stained with a Ponceau‐S solution for 5 min at room temperature and washed twice for 5 min in dH_2_O before scanning.

Furthermore, blots for elucidating the protein content of total‐4E‐BP1 (no. sc‐81149, Santa‐Cruz, Dallas, TX) and p‐mTOR Ser2448 (no. 2971, Cell Signaling Technology, Danvers, MA) were attempted. However, the blots did not give quantifiable protein bands.

### 2.9 Calculation of muscle protein FSR

Muscle myofibrillar protein FSR were calculated according to the precursor‐product method:

$$\mathrm{FSR}=\left[\frac{∆E_{\text{protein}}}{{\hat{E}}_{\text{precusor}}\times ∆t}\right]\times 100$$

where ∆*E*_protein_ is the difference in tracer enrichment between the muscle protein samples, except for the basal period pre‐immobilization where ∆*E*_protein_ is the difference in tracer enrichment between the background plasma protein sample at −160 min (valid in tracer virgins) and the muscle protein sample at 0 min. The *Ê*_precursor_ refers to a surrogate measure of the precursor enrichment, which was calculated based on the weighted average of the plasma enrichments from −150 to 0 min and from 0 to 150 min for the basal and postprandial time periods, respectively. Δ_time_ is the time in hours of tracer exposure between the muscle samples.

### 2.10 Statistics

Before statistical analysis all data sets were tested for normality by evaluating QQ‐plots of residuals. Participant characteristics were analyzed using a two‐way ANOVA with repeated measures for time pre and post immobilization and Holm‐Sidak post hoc tests were performed when significant overall effects were observed. Plasma L‐[*ring*‐^13^C_6_]‐phenylalanine enrichment, and leucine and insulin concentrations were analyzed using a three‐way ANOVA with repeated measures for time pre and post immobilization and for time minutes during the experimental days and Holm‐Sidak post hoc tests were performed when significant overall effects were observed.

Two‐way ANOVA with repeated measures for time pre and post immobilization were conducted on signaling and FSR data. Testing was done on basal, postprandial, and change in FSR from basal to postprandial, respectively, to test the specific hypotheses related to myofibrillar FSR. Signaling data were log‐transformed, normalized to placebo pre immobilization or basal levels at 0 min before ANOVA testing, and shown as the geometric mean (GeoMean) ± back‐transformed standard error mean (SEM). Whenever two‐way ANOVA testing showed significant effect of medication (group) or immobilization (time) (*p* < 0.05), Holm‐Sidak post hoc tests were performed accordingly.

For the quantitative outcome data, the sample mean values are estimates of the population mean and is therefore shown with SEM to indicate the precision of our sample mean. Therefore, data are reported as mean ± SEM with individual data points unless otherwise stated. Statistical significance was considered when *p* < 0.05, and whenever 0.10 > *p* > 0.05 tendencies was discussed. The statistical software GraphPad Prism v. 8.3.1 (GraphPad Software Inc., CA, USA) and Sigma Plot version 13.0 (Systat Software Inc., San Jose, CA, USA) were used for statistical testing.

## 3 RESULTS

### 3.1 Participant characteristic

Age, height, weight, and body mass index did not differ between groups. Body weight (kg) was 78 ± 3 and 79 ± 3 in the Plc group and 83 ± 4 and 83 ± 4 in the Ibu group at baseline and after immobilization, respectively, and did not change significantly with immobilization in both groups.

The participants completed the immobilization period without reporting any clinical problems. Participants reported full compliance regarding the ibuprofen administration and protein intake, which was supported by measurements of blood ibuprofen concentration indicating that all participants in the Ibu group took their medication regularly, as described previously (Dideriksen et al., 2016).

### 3.2 Plasma phenylalanine enrichment

**FIGURE 2:**
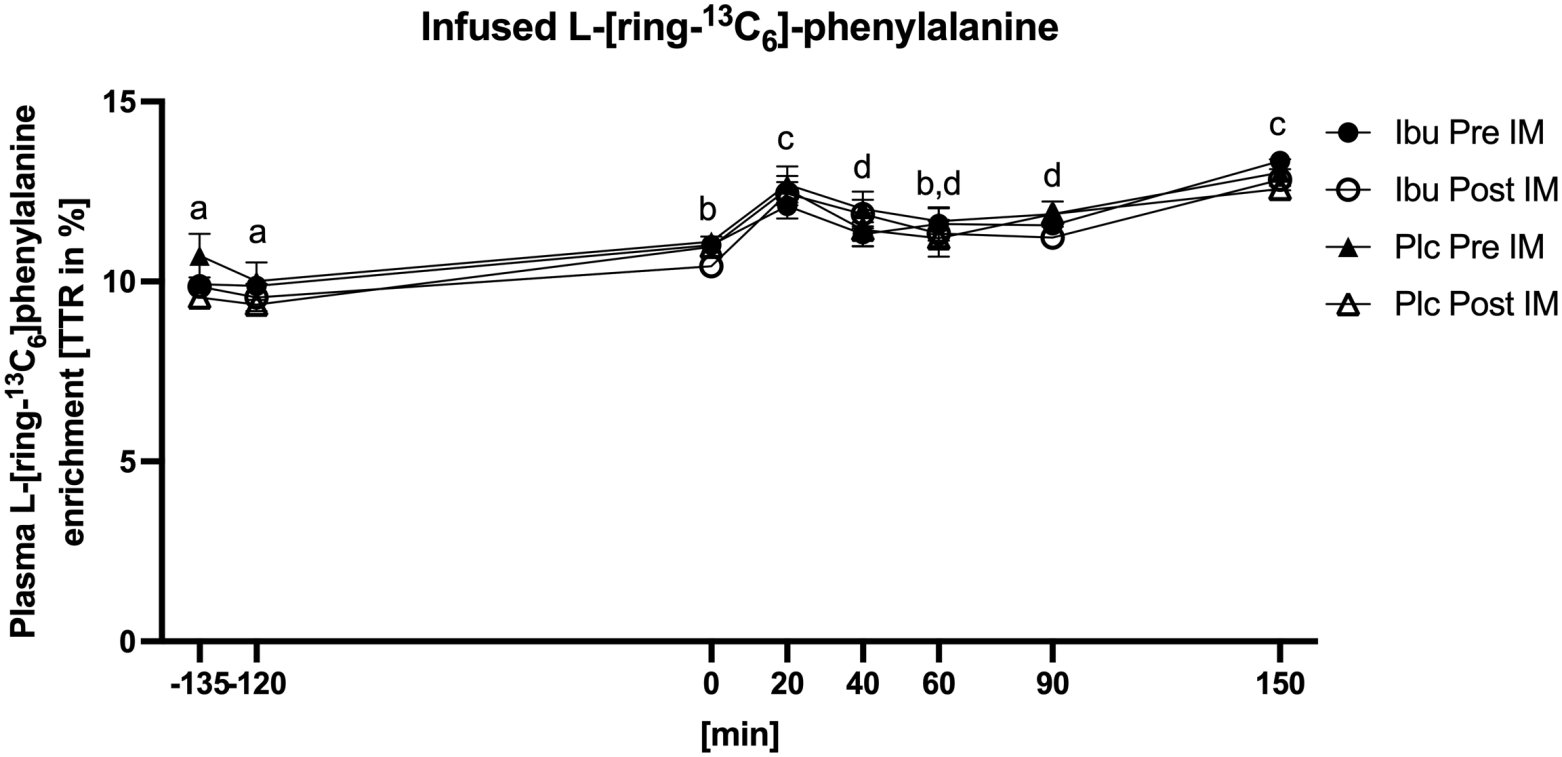
Plasma phenylalanine enrichment measured before (Pre IM) and after (Post IM) 2 weeks of immobilization in participants with ibuprofen (Ibu) or placebo (Plc) administration. Data were analyzed with a three‐way repeated measure ANOVA: A time effect (*p <* 0.0001), but no group (*p* = 0.646) or immobilization effects (*p =* 0.147), were observed. Different letters indicate significant time difference. Data are mean ± SEM.

For plasma enrichment of the infused L‐[*ring*‐^13^C_6_]‐phenylalanine (Figure 2), an overall time effect (*p* < 0.0001) was seen. Post hoc tests indicated that enrichment was lower at −135 and −120 min compared to all other time points (*p*‐values shown in Table S1). Specific significant time differences in period from 0 to 150 min are depicted in Figure 2 and *p*‐values are shown in Table S1. No effects of medication (*p* = 0.646) or immobilization (*p* = 0.147) were found.

### 3.3 Plasma leucine concentration

**FIGURE 3:**
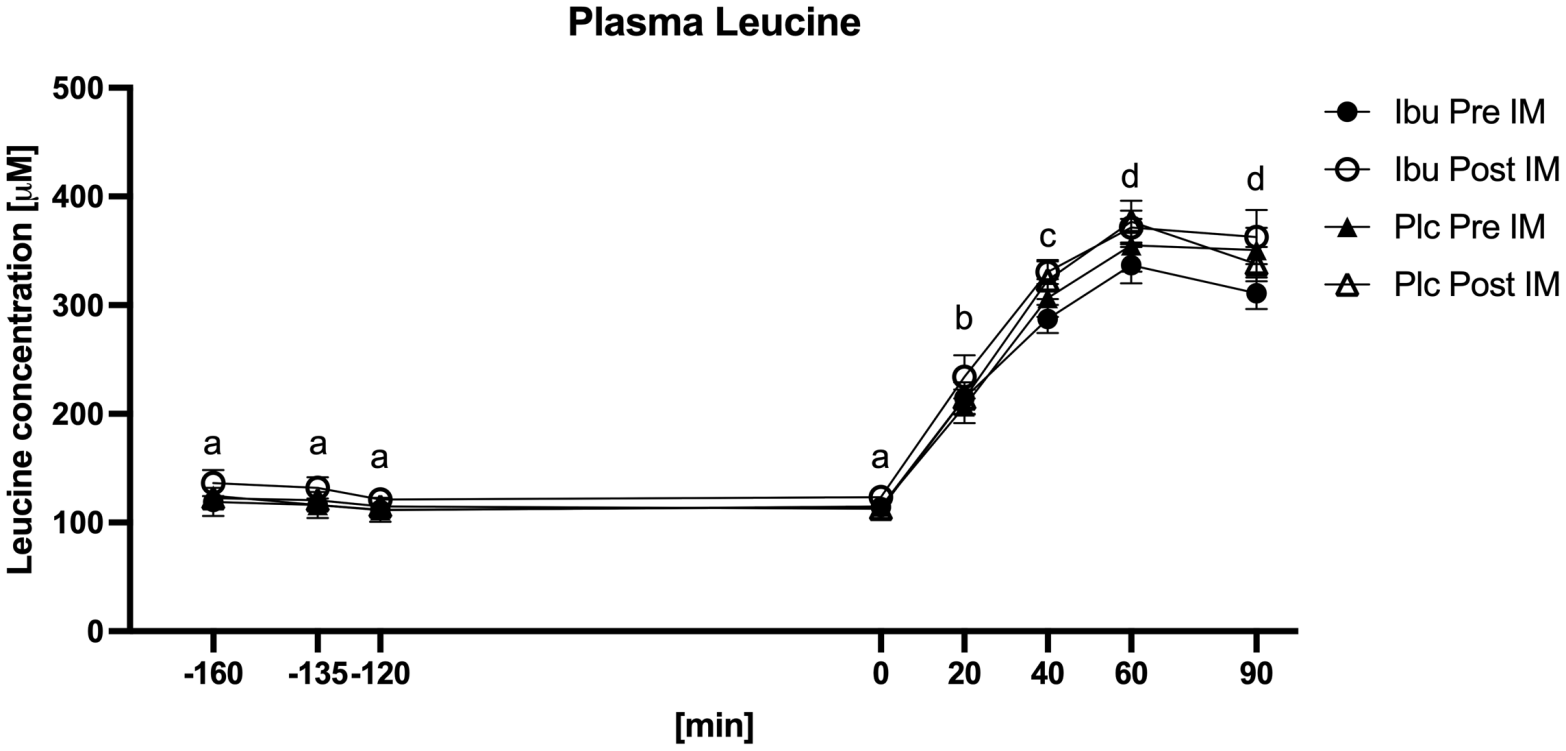
Plasma leucine concentration measured before (Pre IM) and after (Post IM) 2 weeks of immobilization in participants with ibuprofen (Ibu) or placebo (Plc) administration. Data were analyzed with a three‐way repeated measure ANOVA: A time (*p <* 0.0001) and immobilization effect (*p* = 0.028), but no group effect (*p =* 0.938) were observed. Different letters indicate significant time difference. Data are mean ± SEM.

For plasma leucine concentration (Figure 3), there were overall effects of feeding (*p* < 0.0001). Post hoc tests revealed that leucine concentration remained elevated throughout the measured postprandial period (20–90 min after whey protein ingestion) compared to the basal period (the −160‐0 min time interval) (*p‐values* shown in Table S2). At time point 40 min the leucine concentration was higher compared to 20 min, at time point 60 min higher compared to 20 and 40 min, and at time point 90 min higher compared to 20 and 40 min (*p*‐values shown in Table S2).

Moreover, a main effect was found of immobilization (*p* = 0.028), meaning that plasma leucine concentrations were slightly higher after immobilization than before. No medication effect was found (*p* = 0.938).

### 3.4 Plasma insulin concentration

**FIGURE 4:**
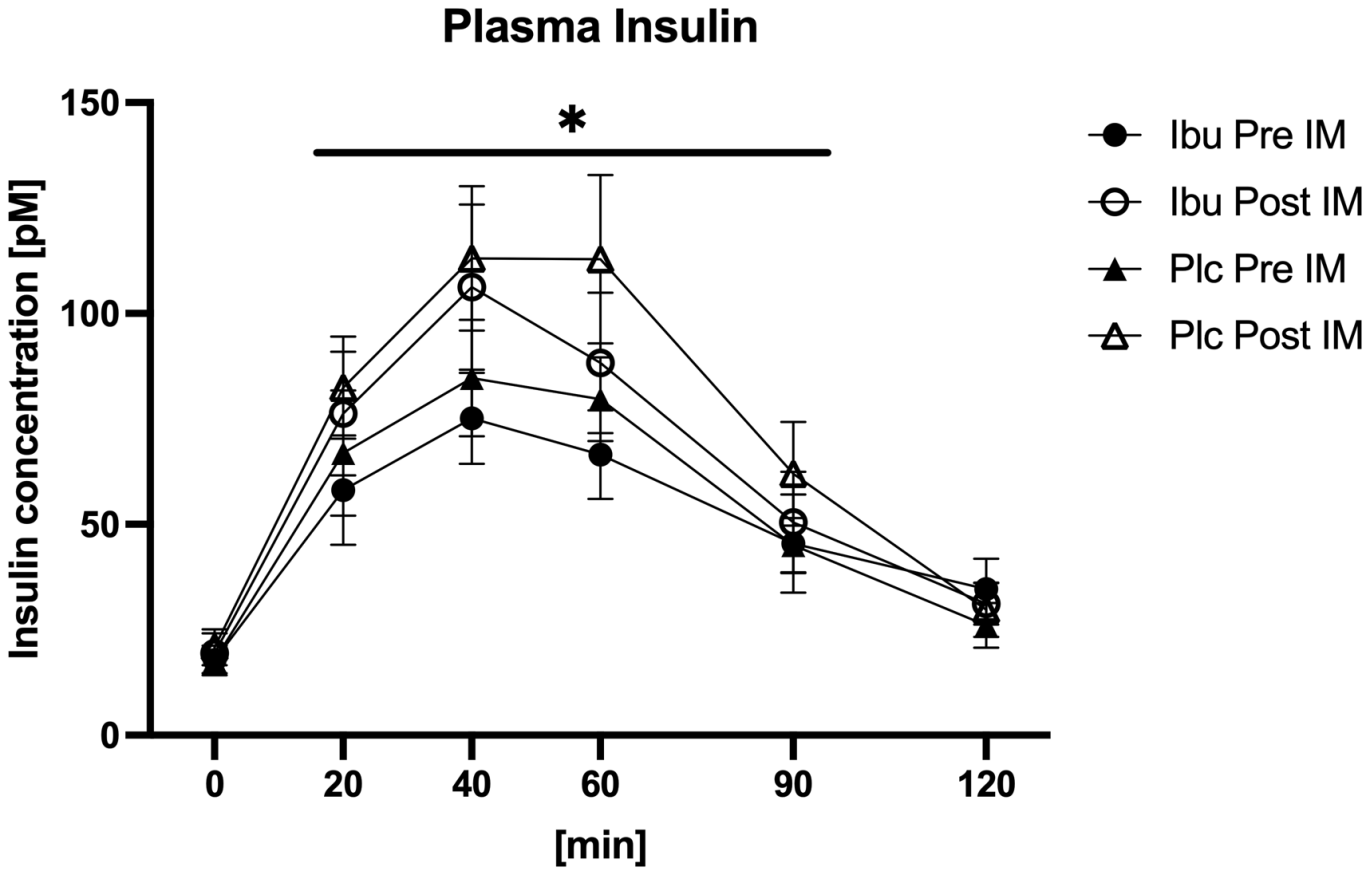
Plasma insulin concentration measured before (Pre IM) and after (Post IM) 2 weeks of immobilization in participants with ibuprofen (Ibu) or placebo (Plc) administration. Data were analyzed with a three‐way repeated measure ANOVA: A time × immobilization interaction was seen (*p* = 0.0007), but no group effect (*p =* 0.653) was observed. * denote time period with different response at pre versus post immobilization. Data are mean ± SEM.

There were a time × immobilization interaction (*p* = 0.0007) and post hoc testing showed that the plasma insulin concentration after whey protein ingestion was higher at 20 (*p* = 0.003), 40 (*p* < 0.0001), 60 (*p* < 0.0001), and 90 min (*p* = 0.027) post compared to pre immobilization period. (Figure 4). All time wise comparisons are shown in Table S3. No effect of medication was found (*p* = 0.653).

### 3.5 Muscle myofibrillar protein synthesis

**FIGURE 5:**
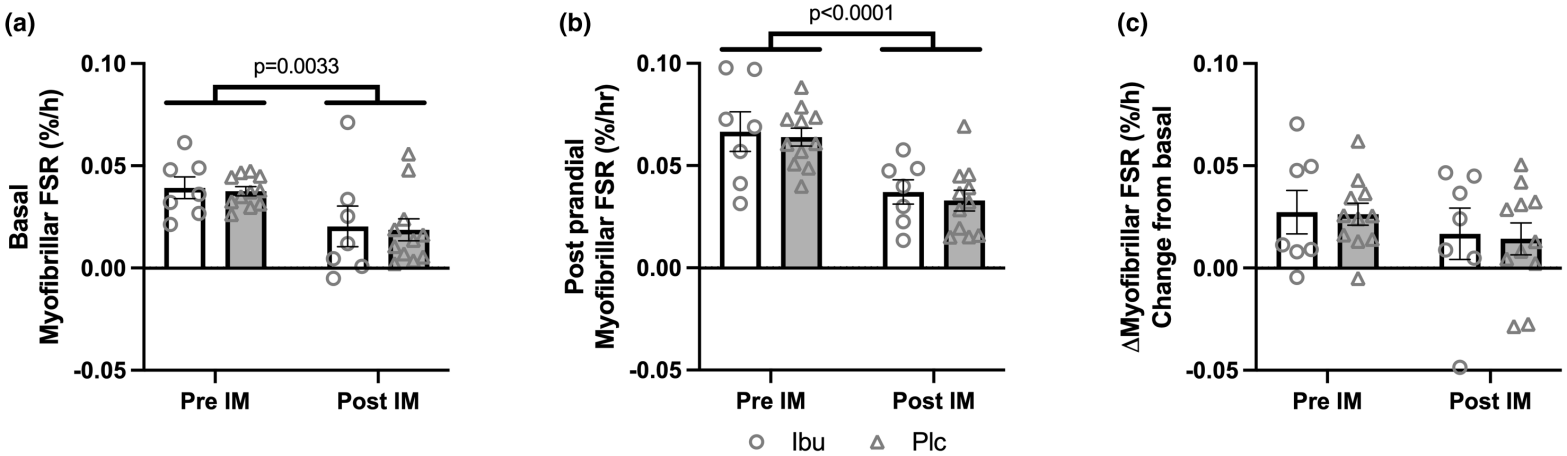
Muscle myofibrillar fractional synthesis rates (FSR) measured before (Pre IM) and after (Post IM) 2 weeks of immobilization in participants with ibuprofen (Ibu) or placebo (Plc) administration. Shown as (a) basal, (b) postprandial, and (c) absolute change from basal to postprandial. Data were analyzed with a two‐way repeated measure ANOVA. Data are shown as mean ± SEM, with individual data points indicated.

Before immobilization, the basal and postprandial myofibrillar FSR values were 0.038 ± 0.002%/h and 0.064 ± 0.004%/h, respectively, in the Plc group and 0.039 ± 0.005%/h and 0.067 ± 0.010%/h, respectively, in the Ibu group. After immobilization, the basal and postprandial FSR values were 0.019 ± 0.005%/h and 0.033 ± 0.005%/h, respectively, in the Plc group and 0.020 ± 0.010%/h and 0.037 ± 0.006%/h, respectively, in the Ibu group. The basal myofibrillar FSR values were significantly lower (*p* = 0.003) after compared to before the immobilization period (Figure 5a). The postprandial myofibrillar FSR was significantly lower (*p* < 0.001) after compared to before immobilization (Figure 5b). However, the absolute changes in myofibrillar FSR from basal to postprandial state were similar (*p* = 0.156) before and after immobilization (Figure 5c). No differences were seen in basal, postprandial, or change (basal to postprandial) in myofibrillar FSR between the Plc and Ibu groups.

### 3.6 Muscle protein synthesis signaling (basal levels pre vs. post immobilization)

To explore the effect of immobilization on overnight fasting (basal) anabolic signaling, all individual's signaling data were normalized to the Plc group mean protein content pre immobilization. This was done also for the subjects in the Ibu group to analyze for any main‐effect of the Ibu intake, since Ibu intake commenced 2 days prior to the pre‐immobilization time point.

**FIGURE 6:**
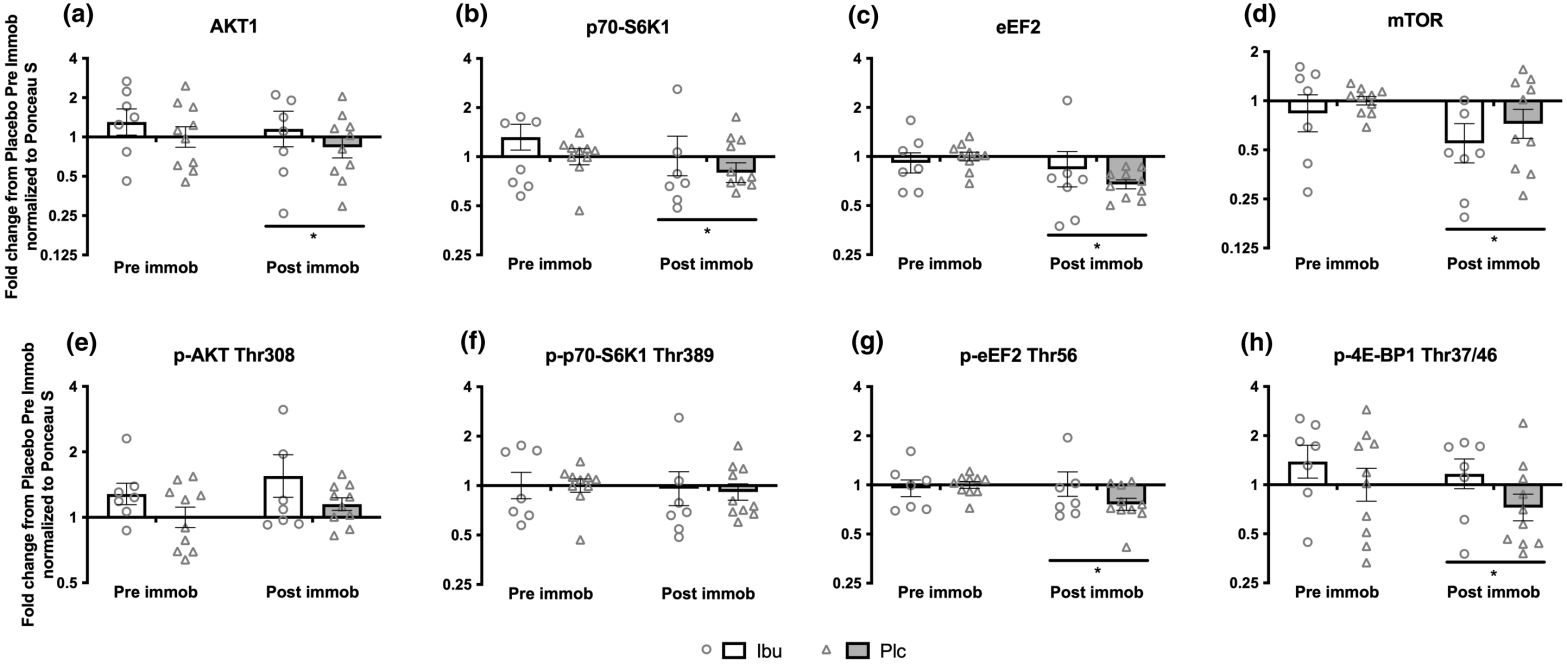
Basal level of select targets from the mTORC1 signaling pathway at pre and post immobilization; total protein content of (a) AKT1, (b) mTOR, (c) p70‐S6K1 and (d) eEF2, as well as phosphorylated protein status of (e) p‐AKT1 Thr308, (f) p‐p70‐S6K1 Thr389 (g) p‐eEF2 Thr56 and (h) p‐4E‐BP1 Thr37/46. Data were normalized to the fasting level at 0 min in the Plc group at pre immobilization. Data are shown as the geometric mean (GeoMean) ± back‐transformed SEM. * denote different from pre immobilization. Representative blots of the quantified protein bands are shown in Figure 8.

The protein content of total‐AKT1 (*p* = 0.0004), total‐mTOR (*p* = 0.004), total‐p70‐S6K1 (*p* = 0.004) and total‐eEF2 (*p* = 0.002) was lower at post‐ compared to pre immobilization with no significant effect of medication (Figure 6a–d). For p‐AKT1 (Thr308) and p‐p70‐S6K1 (Thr389) no significant differences were seen (Figure 6e,f). For p‐eEF2 (Thr56) (p = 0.015) and p‐4E‐BP1 (Thr37/46) (*p* = 0.022) the phosphorylation status was lower at post‐ compared to pre immobilization with no significant effect of medication (Figure 6g,h). Representative blots of the quantified protein bands are shown in Figure 8, and phosphor/total protein ratio of AKT1, p70‐S6K1, and eEF2 are reported in Figure S2.

### 3.7 Muscle protein synthesis signaling (response to protein feeding pre vs. post immobilization)

The response to whey protein feeding on the anabolic signaling was explored by normalizing postprandial signaling data at 150 min post whey protein feeding to 0 min fasting levels within each group at pre and post immobilization, respectively.

**FIGURE 7:**
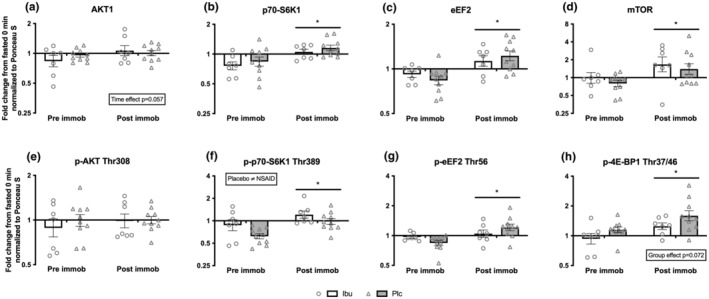
Response to whey protein feeding on select targets from the mTORC1 signaling pathway at pre and post immobilization; total protein content of (a) AKT1, (b) mTOR, (c) p70‐S6K1 and (d) eEF2, as well as phosphorylated protein status of (e) p‐AKT1 Thr308, (f) p‐p70‐S6K1 Thr389 (g) p‐eEF2 Thr56 and (h) p‐4E‐BP1 Thr37/46. Shows signaling data at 150 min post feeding normalized to the fasting level at 0 min at pre and post immobilization, respectively. Data are shown as the geometric mean (GeoMean) ± back‐transformed SEM. * denote different response compared to pre immobilization. Representative blots of the quantified protein bands are shown in Figure 8.

A tendency for an effect of time was seen for total‐AKT1 (*p* = 0.057) (Figure 7a). The protein content at 150 min of total‐mTOR (*p* = 0.012), total‐p70‐S6K1 (*p* = 0.004) and total‐eEF2 (*p* = 0.0003) was greater in response to whey protein feeding at post compared to pre immobilization with no significant differences between groups (Figure 7b–d).

**FIGURE 8:**
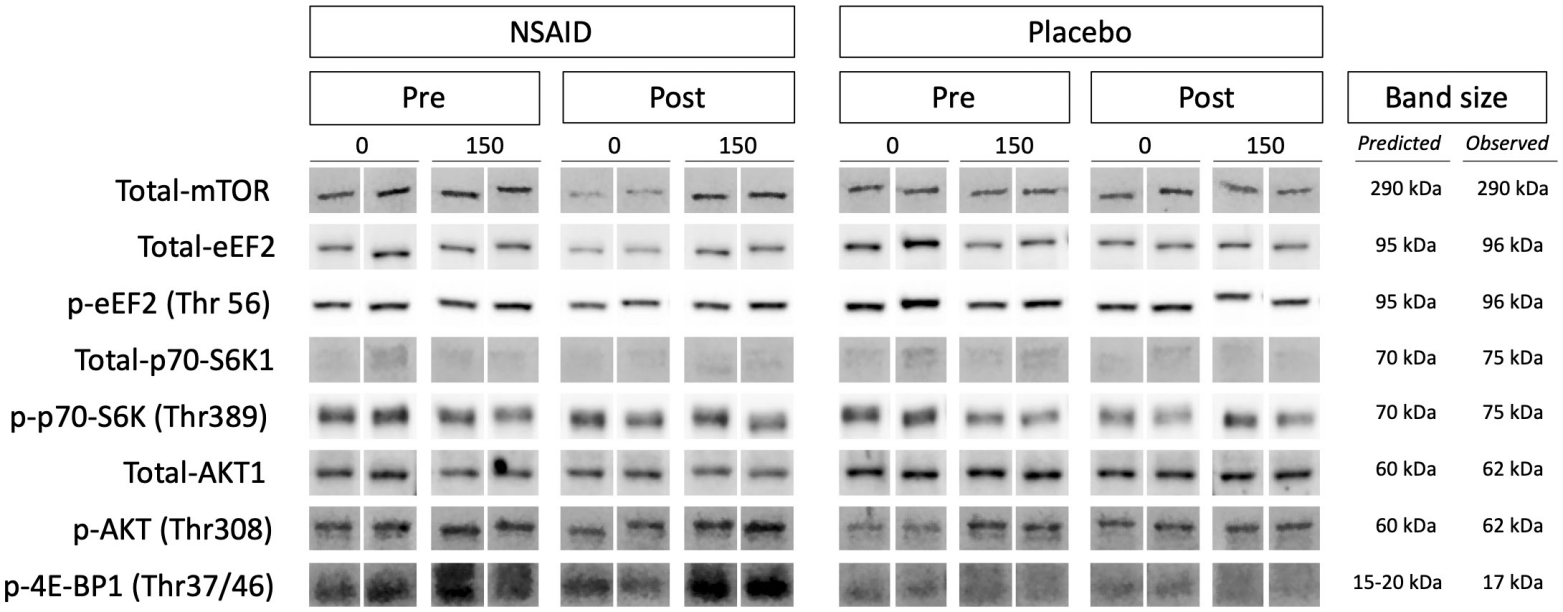
Representative blots from the Western blot analysis of mTOR, eEF2, p‐eEF2(Thr56), p70‐S6K1, p‐p70‐S6K1(Thr389), AKT1, p‐AKT(Thr308), and p‐4E‐BP1(Thr37/46) protein content. Samples were loaded in a randomized order, repeated twice on the same gel. Therefore, images of the bands were reorganized to visually shown them for Ibu (NSAID) and placebo groups, pre and post immobilization, at time point 0 and 150 min. Predicted and observed band sizes are indicated for each target.

No changes were seen in the phosphorylation status of p‐AKT (Thr308), (Figure 7e). The response of p‐p70‐S6K1 (Thr389) (*p* = 0.002), p‐eEF2 (Thr56) (*p* = 0.017) and p‐4E‐BP1 (Thr37/46) (*p* = 0.002) at 150 min post whey protein feeding was greater at post compared to pre immobilization with no significant differences between groups (Figure 7f–h). For p‐p70‐S6K1 (Thr389) the Ibu group had in general a greater phosphorylation status (*p* = 0.034) compared to the Plc group (Figure 7f). A tendency for an effect of group was seen for p‐4E‐BP1 (Thr37/46) (*p* = 0.072) (Figure 7h). Representative blots of the quantified protein bands are shown in Figure 8, and phosphor/total protein ratio of AKT1, p70‐S6K1, and eEF2 are reported in Figure S3.

## 4 DISCUSSION

By applying 2 weeks of single‐leg immobilization this study showed that both fasted and postprandial stimulated myofibrillar FSR was reduced. Interestingly, although the FSR values were reduced after the immobilization period, the absolute anabolic response to whey protein feeding was maintained. After immobilization at the basal state, anabolic signaling protein abundances were decreased and so was the phosphorylation of key anabolic kinases. These basal anabolic signaling protein decrements were circumvented postprandially after immobilization. In contrast to our hypothesis, NSAID treatment did neither affect the myofibrillar synthesis rate in the fasted nor the postprandial state and the anabolic signaling, except for the postprandial P‐p70‐S6K1 (Thr389) phosphorylation, was also unaffected by NSAID treatment.

In accordance with previous findings and our hypothesis, the myofibrillar FSR was markedly reduced after 2 weeks of muscle immobilization in both the fasted and fed state (Figure 5a,b). Similar effects have been shown when applying different muscle unloading regimes and durations (i.e., reduced step count, immobilization, bed rest, and for somewhere between 5 and 21 days) (Breen et al., 2013; de Boer et al., 2007; Drummond et al., 2012; Ferrando et al., 1996; Glover et al., 2008; Kilroe et al., 2020; Wall et al., 2016). However, in contrast to previous studies (Breen et al., 2013; Drummond et al., 2012), we observed a somewhat less degree of anabolic resistance in the present study, as the absolute MPS response to whey protein feeding was maintained after immobilization (Figure 5c). It can be discussed though, whether this is a maintained responsiveness, as the level of postprandial myofibrillar FSR was lower after immobilization (Figure 5b). Yet, a maintained response to protein feeding is supported by the anabolic signaling, which showed a greater feeding response at post versus pre immobilization. This contrasts with the finding by Drummond and colleagues, who found less of a signaling response to essential amino acid (EAA) ingestion after 7 days of bedrest (Drummond et al., 2012). However, in that study an impaired MPS response to EAA ingestion was also observed, which underlines the link between MPS measurements and anabolic signaling. Thus, we are confident that the FSR and anabolic signaling in the current study supports each other and indicate that the current setting of limb immobilization does not induce anabolic resistance.

The present finding mimics previous observations after 5 days of muscle immobilization in young men (Wall et al., 2016). In the previous inactivity studies where more severe anabolic resistance is developed (Breen et al., 2013; Drummond et al., 2012) (i.e., the postprandial stimulation of MPS is markedly impaired, while the postabsorptive MPS is fairly unaffected), whole‐body inactivity models have been applied. It seems likely that whole‐body inactivity regimes may have a more pronounced systemic impact when compared to a local limb immobilization model. Therefore, these contrasting findings may be due to different systemic effects of the muscle unloading regimes; single limb immobilization versus whole‐body inactivity models such as bed rest and reduced step count (Breen et al., 2013; Drummond et al., 2012) and thus, the findings may actually not be contrasting.

In the present study, the participants were older men, who might be slightly more susceptible to the systemic impact of periods of inactivity, such as a higher degree of increased inflammation and insulin resistance development, compared to young individuals. Indeed, the plasma leucine response (Figure 3) to whey protein feeding was higher after compared to before the immobilization period. This finding could indicate that the immobilization period did lead to a reduced leucine uptake into muscle tissue, causing the higher plasma leucine concentration post immobilization. In line with that, the plasma insulin response (Figure 4) to whey protein feeding was also higher after compared to before the immobilization period. It seems possible that the higher insulin response, at least to some extent, was caused by the higher plasma leucine concentration observed post immobilization. Taken together, the higher plasma leucine and insulin concentrations could indicate some degree of systemic insulin resistance after the inactivity period. Previously, it has been shown that 2 weeks of reduced ambulatory activity leads to increased plasma levels of C‐reactive protein (CRP) and tumor necrosis factor alpha (TNF‐α) in older humans (Breen et al., 2013), whereas similar findings of increased systemic inflammation could not be observed in young subjects (Krogh‐Madsen et al., 2010). This supports the notion that periods of muscle inactivity may have more severe consequences in older than in young individuals. On the other hand, the plasma CRP, IL‐6, and TNF‐α levels were all unaffected by the present immobilization period (Dideriksen et al., 2016). This could indicate that the model of reduced ambulatory activity used by Breen et al. (Breen et al., 2013) may result in more severe systemic changes than the present immobilization model, where increased upper‐body activity was required for locomotion.

Previous work in older individuals has displayed increased muscle expression of the inflammatory mediators, IL‐6 and NF‐κB (but not TNF‐α), during and after 2 weeks of lower limb‐immobilization (Suetta et al., 2012). This could suggest that the present inactivity model would induce some degree of local muscle inflammatory response. However, the muscle expression of the TNF‐α and IL‐6 cytokines were unaffected by the present immobilization period and NSAID treatment (Dideriksen et al., 2016). The unchanged systemic and local muscle inflammation levels, might, at least to some extent, explain the lack of anabolic resistance development during the muscle inactivity period. Further, this might be part of the underlying reason explaining why NSAID treatment did not affect the level of inflammatory markers (Dideriksen et al., 2016), and possibly also did not affect the MPS after immobilization. Except for the general greater phosphorylation of p70‐S6K1 in the NSAID group, the lack of impact of NSAID treatment on the postprandial MPS after immobilization contrasted with the study hypothesis.

One limitation of the present study design is that all participants were treated with 75 mg acetylsalicylic acid per day during the 2‐week immobilization period. This was done to reduce the potential risk of deep venous thrombosis. Recent findings indicate that low‐dose aspirin can reduce muscle PGE2 production *ex vivo* (Fountain et al. 2020). However, the potential *in vivo* anti‐inflammatory effect of such a low dose has not been clearly shown and therefore remains unknown.

Another limitation of the present study was the lack of a measurable increase in systemic and local muscle inflammation during immobilization (Dideriksen et al., 2016), which might be caused by the less severe unilateral immobilization model where participants were requested to remain physically active during the immobilization period. In relation to this, the NSAID treatment did not affect the systemic and local muscle inflammation, which might be related to the fairly low level of systemic inflammatory observed in all participants (Dideriksen et al., 2016).

## 5 CONCLUSION

The myofibrillar synthesis rate was reduced after the immobilization period both in the post‐absorptive basal state and postprandial state. However, even though the MPS was reduced, the anabolic response to whey protein feeding was maintained in absolute terms. NSAID treatment had no impact on myofibrillar protein synthesis adaptations to immobilization. Finally, anabolic signaling differed between the measured time points, but, correspondingly, the signaling was unaffected by NSAID treatment.

## AUTHOR CONTRIBUTIONS

K.D., M.K., and L.H. designed study; K.D., A.P.B., and L.H. conducted the experimental work; K.D., S.R., J.A., M.Z., and L.H. analyzed and interpreted data; K.D. drafted manuscript; K.D., S.R., J.A., M.Z., A.P.B., M.K., and L.H. edited and revised manuscript. All authors approved the final content of the manuscript.

## FUNDING INFORMATION

Funding is greatly acknowledged from: Danish Dairy Research Foundation, Danish Council for Independent Research (09‐073587), and Center for Healthy Aging (Nordea Foundation). Arla Foods Ingredients P/S provided the Lacprodan whey protein. The funding sources were not involved in the preparation or completion of the study or in the writing of the manuscript.

## CONFLICT OF INTEREST STATEMENT

The authors declare no conflicts of interest and there are no financial conflicts to disclose.

## ETHICS STATEMENT

All participants gave their written informed consent before being enrolled in the experiment that was approved by the Copenhagen Ethics Committee (H‐1‐2010‐007) and conformed to the Helsinki Declaration.

## Supporting information


Data S1:

## Data Availability

The data presented in this study are available on request from the corresponding author.
