# Supplementary material for: Lower basal and postprandial muscle protein synthesis after 2 weeks single‐leg immobilization in older men: No protective effect of anti‐inflammatory medication
Source: Physiol Rep. 2024 Feb 26;12(4):e15958. doi: 10.14814/phy2.15958 (PMC10895449; doi:10.14814/phy2.15958)
Supplement: Supplementary file 1 — Data S1: [file PHY2-12-e15958-s001.pdf]

Supplementary material

Holm-Šídák's multiple comparisons test	Adjusted *p*-value
-135 vs. -120	0,6045
-135 vs. 0	0,0068
-135 vs. 20	<0,0001
-135 vs. 40	<0,0001
-135 vs. 60	<0,0001
-135 vs. 90	<0,0001
-135 vs. 150	<0,0001
-120 vs. 0	<0,0001
-120 vs. 20	<0,0001
-120 vs. 40	<0,0001
-120 vs. 60	<0,0001
-120 vs. 90	<0,0001
-120 vs. 150	<0,0001
0 vs. 20	<0,0001
0 vs. 40	0,0159
0 vs. 60	0,1230
0 vs. 90	0,0192
0 vs. 150	<0,0001
20 vs. 40	0,0159
20 vs. 60	0,0012
20 vs. 90	0,0123
20 vs. 150	0,2101
40 vs. 60	0,7844
40 vs. 90	0,9099
40 vs. 150	<0,0001
60 vs. 90	0,7844
60 vs. 150	<0,0001
90 vs. 150	<0,0001

Supplementary table S1. An overall time-effect of L-[ring-$^{13}C_6$]-phenylalanine enrichment was seen. Subsequent Holm-Sidak posthoc testing was performed. The *p*-values are shown for the specific time comparisons.

Holm-Šídák's multiple comparisons test	Adjusted *p*-value
-160 vs. -135	0,9500
-160 vs. -120	0,8678
-160 vs. 0	0,8860
-160 vs. 20	<0,0001
-160 vs. 40	<0,0001
-160 vs. 60	<0,0001
-160 vs. 90	<0,0001
-135 vs. -120	0,9500
-135 vs. 0	0,9500
-135 vs. 20	<0,0001
-135 vs. 40	<0,0001
-135 vs. 60	<0,0001
-135 vs. 90	<0,0001
-120 vs. 0	0,9500
-120 vs. 20	<0,0001
-120 vs. 40	<0,0001
-120 vs. 60	<0,0001
-120 vs. 90	<0,0001
0 vs. 20	<0,0001
0 vs. 40	<0,0001
0 vs. 60	<0,0001
0 vs. 90	<0,0001
20 vs. 40	<0,0001
20 vs. 60	<0,0001
20 vs. 90	<0,0001
40 vs. 60	<0,0001
40 vs. 90	0,0395
60 vs. 90	0,3283

Supplementary table S2. An overall time-effect of plasma leucine concentration was seen. Subsequent Holm-Sidak posthoc testing was performed. The *p*-values are shown for the specific time comparisons.

Holm-Šídák's multiple comparisons test	Adjusted *p*-value
Time point 0 min	
Pre immob vs. Post immob	0,6444
Time point 20 min	
Pre immob vs. Post immob	0,0030
Time point 40 min	
Pre immob vs. Post immob	<0,0001
Time point 60 min	
Pre immob vs. Post immob	<0,0001
Time point 90 min	
Pre immob vs. Post immob	0,0265
Time point 120 min	
Pre immob vs. Post immob	0,8665
Pre immob	
0 vs. 20	<0,0001
0 vs. 40	<0,0001
0 vs. 60	<0,0001
0 vs. 90	<0,0001
0 vs. 120	0,1009
20 vs. 40	0,0086
20 vs. 60	0,1009
20 vs. 90	0,0068
20 vs. 120	<0,0001
40 vs. 60	0,2366
40 vs. 90	<0,0001
40 vs. 120	<0,0001
60 vs. 90	<0,0001
60 vs. 120	<0,0001
90 vs. 120	0,0173
Post immob	
0 vs. 20	<0,0001
0 vs. 40	<0,0001
0 vs. 60	<0,0001
0 vs. 90	<0,0001
0 vs. 120	0,1309
20 vs. 40	<0,0001
20 vs. 60	0,0002
20 vs. 90	0,0002
20 vs. 120	<0,0001
40 vs. 60	0,1951
40 vs. 90	<0,0001
40 vs. 120	<0,0001
60 vs. 90	<0,0001
60 vs. 120	<0,0001
90 vs. 120	<0,0001

Supplementary table S3. An overall time x immobilization effect of plasma insulin concentration was seen. Subsequent Holm-Sidak posthoc testing was performed. The *p*-values are shown for the time x immobilization comparisons.

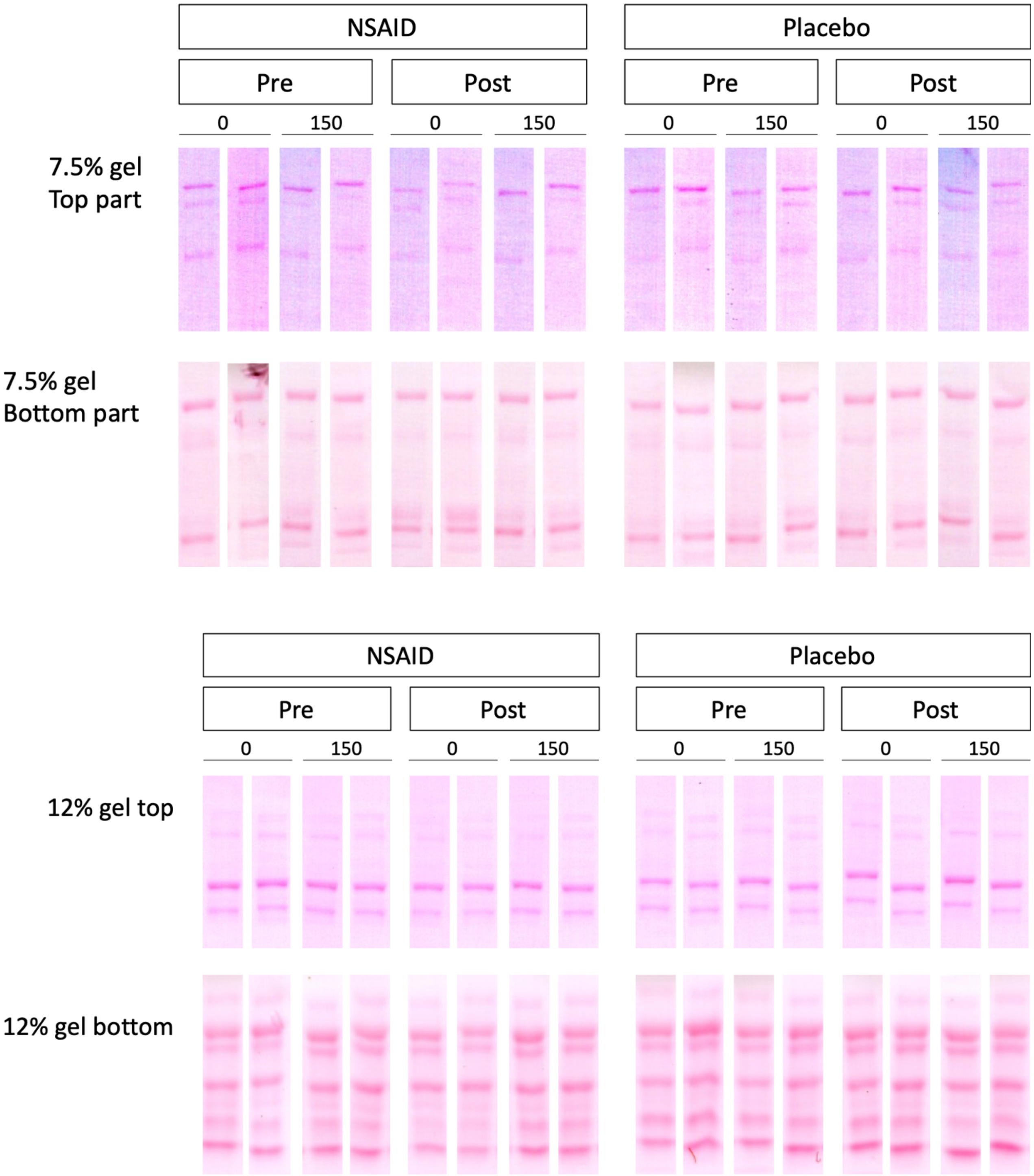

Supplementary figure 1. Representative blots of Ponceau S staining of 7.5% and 12% SDS-PAGE gels respectively. Top and bottom sections of the gels were cut, in order to fit three top or bottom pieces on one membrane, to reduce the number of membranes that were analyzed for the total and phosphorylated protein content of mTOR (7.5% top), p70-S6K1 (7.5% bottom), eEF2 (12% top), AKT1 (12% bottom), 4E-BP1 (12% bottom). Samples were loaded in a randomized order, repeated twice on the same gel. Therefore, images of the bands were reorganized to visually shown them for Ibu (NSAID) and placebo groups, pre and post immobilization, at time point 0 and 150 min.

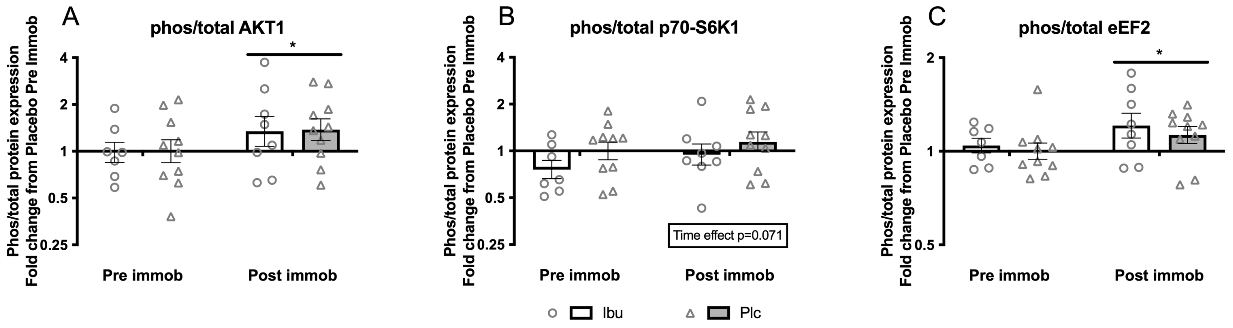

Supplementary figure 2 Effect of immobilization on the basal level of select targets from the mTORC1 signaling pathway; phosphorylated to total protein ratio of A) p-AKT1 Thr308/total AKT1, B) p-p70-S6K1 Thr389/total p70-S6K1 and C) p-eEF2 Thr56/total eEF2. Data were normalized to the fasting level at 0 min in the Plc group at pre immobilization. Data are shown as the geometric mean (GeoMean) ± back-transformed SEM. * denote different from pre immobilization.

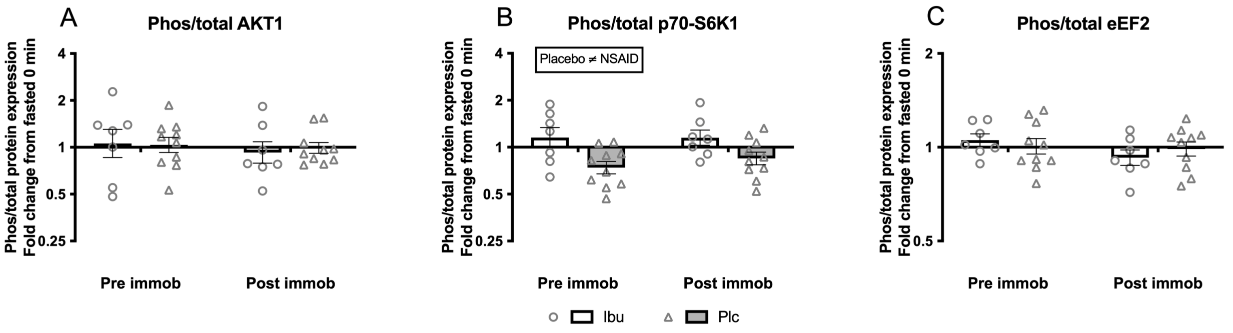

Supplementary figure 3 Response to whey protein feeding on select targets from the mTORC1 signaling pathway; phosphorylated to total protein ratio of A) p-AKT1 Thr308/total AKT1, B) p-p70-S6K1 Thr389/total p70-S6K1 and C) p-eEF2 Thr56/total eEF2. Shows signaling data at 150 min post feeding normalized to the fasting level at 0 min. Data are shown as the geometric mean (GeoMean) ± back-transformed SEM.
